# Advanced glaucoma following Nd:YAG capsulotomy: a case report

**DOI:** 10.1186/s12886-018-0852-9

**Published:** 2018-09-14

**Authors:** Colin Goudie, Andrew Tatham

**Affiliations:** Princess Alexandra Eye Pavilion, Edinburgh, Scotland

**Keywords:** Pupil block, Aphakia, YAG capsulotomy, Secondary glaucoma

## Abstract

**Background:**

We present a case of aphakic pupil block caused by vitreous prolapse into the anterior chamber following Nd:YAG capsulotomy.

**Case presentation:**

This resulted in advanced glaucoma in a young patient, which presented a significant clinical management challenge.

**Conclusions:**

Ultimately, at the time of writing, her intraocular pressure and uveitis were well controlled, however the long-term outcome remains uncertain, given the uncompromising natural history of her complicated ocular condition.

## Background

A 22-year-old female was referred to the glaucoma clinic with uncontrolled intraocular pressure and advanced cupping of the right optic disc. She was referred by the local uveitis clinic with an extensive past ophthalmic history. Growing up, she was under the care of another ophthalmology department, having been diagnosed with pauciarticular juvenile idiopathic arthritis (JIA) at the age of ten soon before developing chronic anterior uveitis in the right eye. She required no immunosuppression for her JIA and was discharged from the rheumatologists aged 18. Throughout her teenage years she was treated with intermittent topical steroid drops. From the age of 13–16 she was treated with tacrolimus and aged 18 and 19 she required two course of oral prednisolone to control her uveitis. At the age of 18 she had a cataract removed from the right eye and was left aphakic before having a right Nd:YAG laser capsulotomy 8 months later to treat posterior capsule opacification. After this procedure she developed raised intraocular pressure (IOP) in the right eye. When she moved to university she was referred to our local uveitis clinic.

**Fig. 1:**
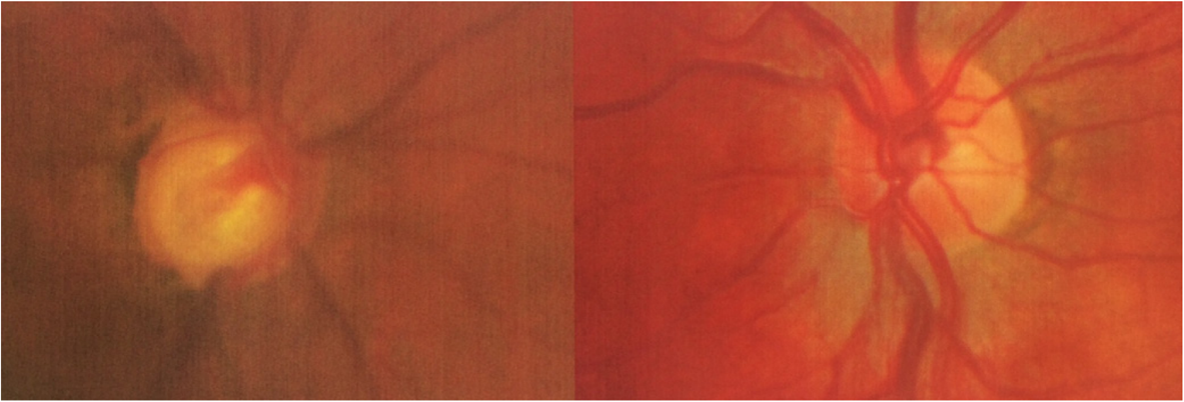
Optic disc photos showing at presentation to the uveitis clinic, showing advanced cupping in the right eye

At this stage her visual acuity (corrected with a pinhole) was 1/36 in the right eye and 6/4 in the left. Her IOP was 24 in the right and 15 in the left and her only medication was dorzolamide eye drops twice a day in the right eye. Her right optic disc was significantly cupped (see Fig. 1) and she had a large right sensory exotropia. She was started on topical acular three times daily to the right eye as well as dorzolamide twice a day. She was also seen by the orthoptists who felt that a cosmetic contact lens was the best management of her exotropia (squint surgery was unlikely to be successful due to poor fusional control and an element of torsion).

Over the next 2 years she had no significant flares of uveitis, however her IOP in the right eye remained poorly controlled. Throughout this time her vision in the right eye deteriorated to hand movements (HM). Of note she had previously been tried on topical beta-blocker but did not tolerate this due to lethargy. She was started on topical brimonidine but did not tolerate this due to stinging. She was treated with a Nd:YAG peripheral iridotomy which yielded no significant improvement in her IOP. At this stage she was referred to the glaucoma clinic.

## Case presentation

**Fig. 2:**
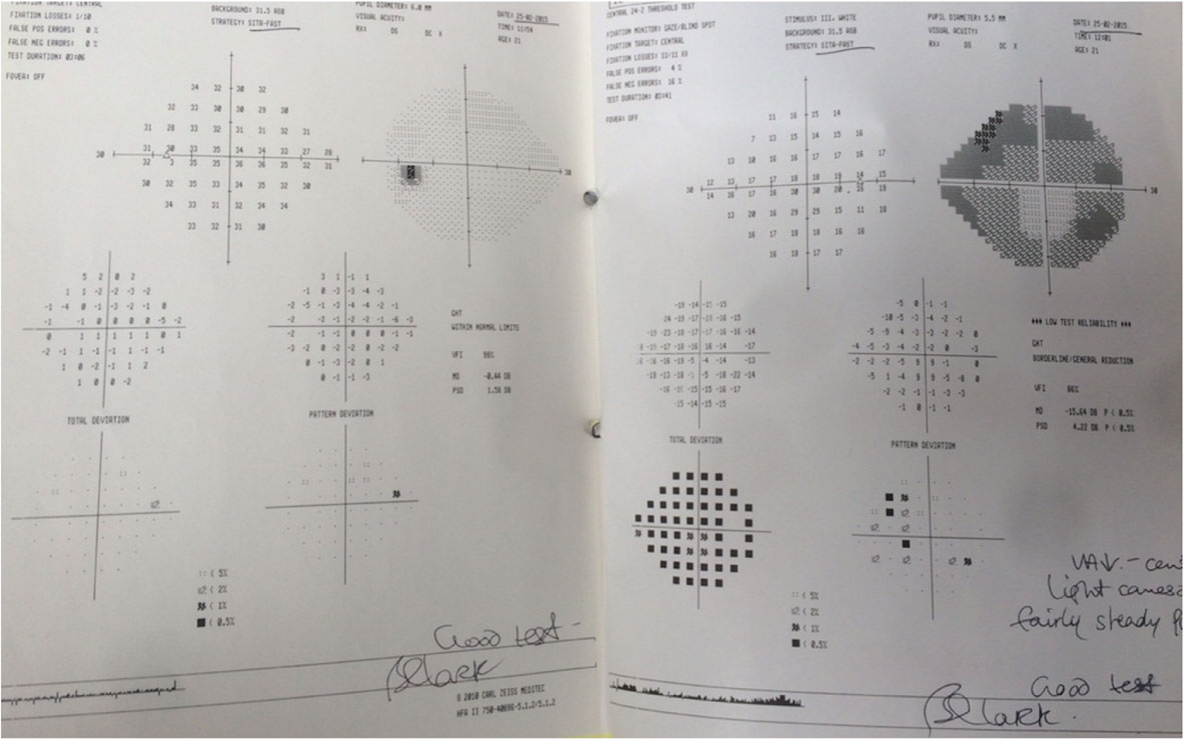
Visual fields at the first appointment in the glaucoma clinic showing advanced field loss in the right eye

**Fig. 3:**
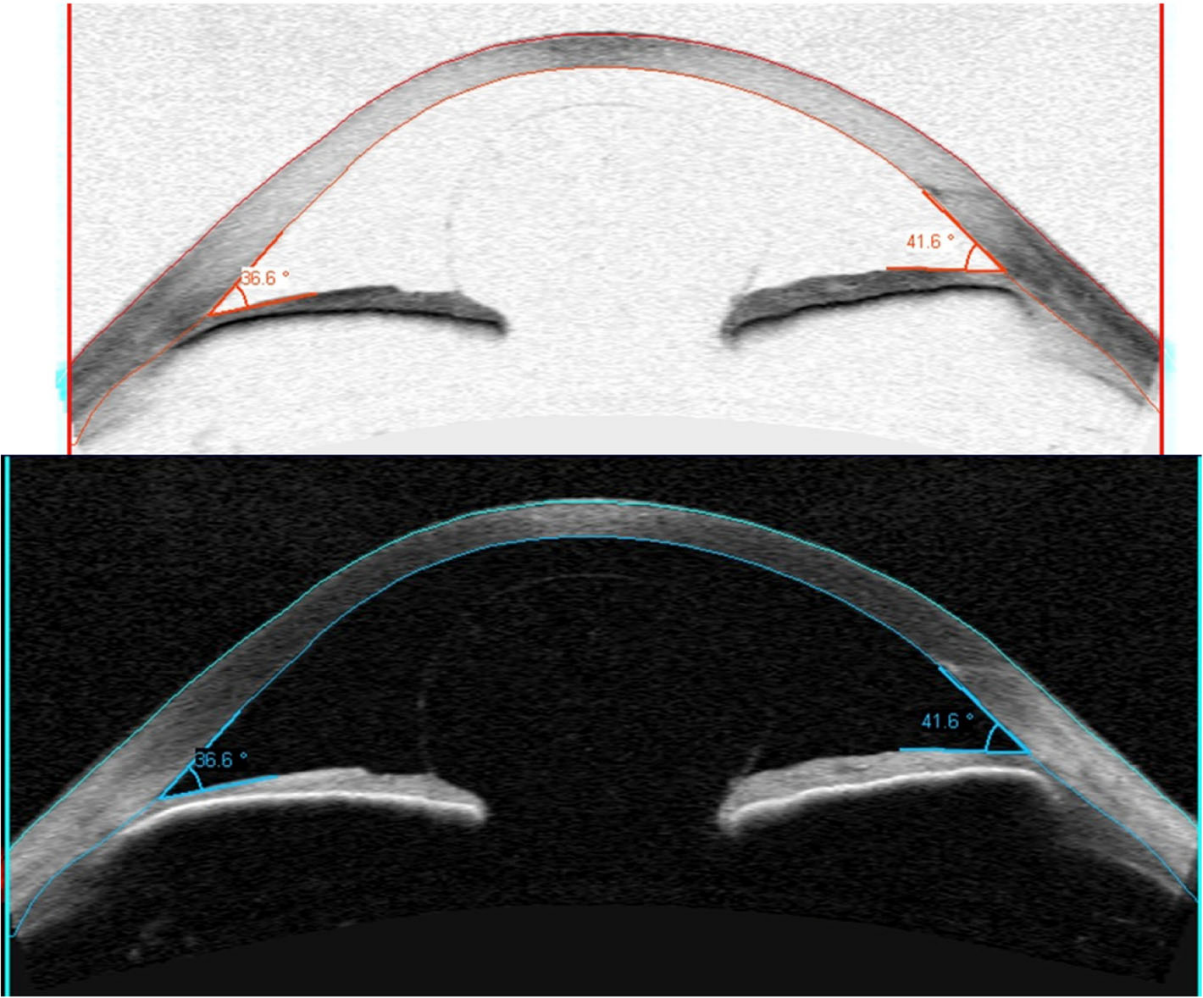
Anterior segment OCT pictures showing vitreous prolapse through the pupil into the anterior chamber of the right eye

The patient was not particularly concerned about the poor vision in her right eye because this was not new and over the years she had learnt to adapt. Her eyes were not uncomfortable and she did not suffer from headaches. Visual acuity was HM in the right eye and 6/4 in the left. Her IOP was measured at 32 in the right eye and 8 in the left. OCT of the retinal nerve fibre layers confirmed advanced thinning in the right eye. Visual fields are shown in Fig. 2. Central corneal thickness was measured at 496 μm in the right eye and 471 μm in the left. Anterior segment examination showed vitreous prolapsing through the pupil into the anterior chamber and gonioscopy showed almost 360 degrees of peripheral anterior synachiae with no bombe. This is impressively shown on an anterior segment OCT scan in Fig. 3.

### Management

The underlying explanation for her intractable elevation of IOP was aphakic pupil block, which developed after Nd:YAG capsulotomy. Despite the eye’s poor visual prognosis, the importance of controlling the IOP was emphasised to the patient. Despite her lack of new symptoms, we recommended that additional treatment was required to avoid further visual field loss and other long-term complications of elevated IOP (such as vein occlusion). We discussed the potential difficulties of controlling her IOP, given her history of anterior uveitis, aphakia and mechanical pupil block. She was tentatively started on preservative free latanoprost, however this yielded no significant improvement. She was eventually treated with cyclodiode laser and a post-operative course of topical maxidex. Three months after treatment her IOP has remained well controlled, most recently 14 mmHg, on topical dorzolamide and latanoprost preservative free. There has been no recurrence of anterior uveitis.

## Discussion and conclusions

We have reported a case of aphakic pupil block glaucoma following Nd:YAG capsulotomy, which was very nicely illustrated using anterior segment OCT. This case highlighted a number of difficulties in managing uveitis and its sequelae in young patients. Our patient required multiple medical and surgical treatments, ultimately creating a complicated clinical picture, which provided a significant challenge. Persistently raised intraocular pressure resulted in irreversible optic nerve head damage and despite good IOP control following her most recent intervention the long-term outcome remains uncertain.

Pupil block is increased resistance to the flow of aqueous from posterior chamber through the pupil to the anterior chamber, leading to anterior bowing of the peripheral iris, which blocks off the aqueous drainage through the trabecular meshwork. The most common cause is the crystalline lens, as seen in acute angle closure. Other causes include the lens capsule (which is rare with modern surgery techniques), intraocular lens or vitreous. The anterior segment OCT images very nicely illustrate prolapse of the intact anterior hyaloid surface, resulting in pupil block.

Modern cataract surgery, with IOL implantation into an intact posterior capsular bag, means that there are very few recent reported cases of post-operative pupil block. An intact capsular bag and an implanted IOL both act as barriers between the anterior and posterior chambers and, if the anterior vitreous face is intact, there are no aggravating factors to initiate a prolapse. Older methods of lens extraction surgery resulted in higher rates of aphakia and disruption of the posterior lens capsule and anterior vitreous face. A case series from 1979 reported 7 cases and described a surgical approach to its management [1].

In our case pupil block was caused by Nd:YAG capsulotomy, which resulted in vitreous prolapse into the anterior chamber. Nd:YAG capsulotomy is a non-invasive, safe treatment of posterior capsular opacification [2–4]. Complications of this procedure include IOL movement or damage, cystoid macular oedema and retinal tear or detachment. Whilst transient anterior chamber inflammation has been reported, persistant iritis or vitritis is rare [5]. The most common complication is a spike in IOP 2–4 h after the procedure, which is reported in 15–30% of patients (despite prophylactic treatment) and is more pronounced in patients with pre-existing glaucoma. A recent paper described loss of IOP control secondary to bleb fibrosis, following Nd:YAG laser capsulotomy in trabeculectomised eyes with previously well functioning filtering blebs [6]. Pupil block following Nd:YAG capsulotomy is rare, with data in the literature limited to case reports [3, 7].

Aphakic pupil block with advanced optic disc cupping secondary to raised IOP in a patient with previous anterior uveitis presents a significant management dilemma. By the time our patient attended the glaucoma clinic, use of all topical IOP lower agents has been exhausted. She was previously intolerant of brimonidine and timolol, while her IOP was inadequately controlled on dorzolamide. The use of prostaglandin analogues remains controversial in patients with uveitis due to their theoretical pro-inflammatory mechanism of action [8]. In our case presevative free latanoprost was cautiously started, which caused no increase in anterior chamber inflammation, however there was also no significant improvement in her IOP.

Laser peripheral iridotomy (LPI) was also unsuccessful. LPIs create an alternative channel for fluid to flow between the anterior and posterior chambers and can prevent or eliminate pupil block and deepen the anterior chamber. They are the standard therapeutic modality for treatment of patients at risk of primary angle closure, however in our case, LPI was unable to overcome the mechanical pupil block caused by vitreous prolapse.

Surgery in any eye with uveitis carries significant risk of inflammation, with higher risk of poor response to treatment, hypotony and psthisis [9]. Trabeculectomy was excluded, due to the high rate of failure in uveitis as well as increased risk of postoperative complications [10]. Anterior vitrectomy, pars plan vitrectomy and alternative glaucoma surgery, such as a glaucoma drainage device or iStent, were considered, however given the eye’s poor visual potential, atypical anatomy and complicated history the decision was made to avoid invasive surgery.

Another, somewhat attractive option in this case, was to do nothing. Given the significant progression of her nerve fibre layer thinning and field defect this would have almost certainly result in further visual field loss. Moreover, uncontrolled IOP carries a significant risk of central retinal vein occlusion, opening the door to the associated significant complications, such as rubeiotic glaucoma, which would result in a painful, non-seeing eye.

After much discussion with the patient the decision was made to perform transscleral diode laser cyclophotocoagulation (cyclodiode). The literature provides conflicting evidence regarding the effectiveness of cyclodiode in patients with uveitis. There are two studies focusing on refractory glaucoma caused by inflammatory eye disease. The first, which included patients of all ages reported a success rate of 72.2% [11]. The other study looked at the outcomes of in children with chronic anterior uveitis secondary to JIA. The mean age in their cohort was 10.9 (range 6.2–19.5). They found success rate of 32% at 10.1 months [12]. The remaining studies on cyclodiode included only a few inflammatory glaucoma patients, with an overall success rate of 66–81% although of all the eyes included in each study, only a few suffered from uveitis. In two studies, eyes with uveitis did not respond differently to other groups (each consisting of nine eyes with uveitis) [13, 14]. In another study in adults, success was obtained in two of five eyes with uveitic glaucoma [15].

Another complicating factor in our case is aphakia, which is associated with an increased risk of complications following cyclodiode, including retinal detachment or phthisis bulbi [16, 17].

Ultimately, her intraocular pressure and uveitis seem well controlled, however the long-term outcome remains uncertain, given the uncompromising natural history of her complicated ocular condition. The anterior segment OCT pictures impressively illustrate an unusual cause of secondary glaucoma whilst the clinical story highlights the difficulties in managing patients with multiple ocular co-morbidities.
